# Effect of the Enhanced Production of Chlorophyll *b* on the Light Acclimation of Tomato

**DOI:** 10.3390/ijms24043377

**Published:** 2023-02-08

**Authors:** Imran Khan, Ahmad Zada, Ting Jia, Xueyun Hu

**Affiliations:** 1 International Research Laboratory of Agriculture and Agri-Product Safety of the Ministry of Education of China, Yangzhou University, Yangzhou 225009, China; 2 Laboratory of Plant Functional Genomics of the Ministry of Education, Yangzhou University, Yangzhou 225009, China; 3 College of Bioscience and Biotechnology, Yangzhou University, Yangzhou 225009, China

**Keywords:** tomato (*Solanum lycopersicum* Mill.), chlorophyll *b*, chlorophyllide *a* oxygenase, high-light, low-light, reactive oxygen species (ROS)

## Abstract

Tomato (*Solanum lycopersicum* Mill.) is one of the widely cultured vegetables under protected cultivation, in which insufficient light is one of the major factors that limit its growth, yield, and quality. Chlorophyll *b* (Chl *b*) is exclusively present in the light-harvesting complex (LHC) of photosystems, while its synthesis is strictly regulated in response to light conditions in order to control the antenna size. Chlorophyllide *a* oxygenase (CAO) is the sole enzyme that converts Chl *a* to Chl *b* for Chl *b* biosynthesis. Previous studies have shown that overexpressing CAO without the regulating domain (A domain) in Arabidopsis overproduced Chl *b*. However, the growth characteristics of the Chl *b* overproduced plants under different light environmental conditions are not well studied. Considering tomatoes are light-loving plants and sensitive to low light stress, this study aimed to uncover the growth character of tomatoes with enhanced production of Chl *b*. The A domain deleted Arabidopsis CAO fused with the FLAG tag (BCF) was overexpressed in tomatoes. The BCF overexpressed plants accumulated a significantly higher Chl *b* content, resulting in a significantly lower Chl *a/b* ratio than WT. Additionally, BCF plants possessed a lower maximal photochemical efficiency of photosystem II (Fv/Fm) and anthocyanin content than WT plants. The growth rate of BCF plants was significantly faster than WT plants under low-light (LL) conditions with light intensity at 50–70 µmol photons m^−2^ s^−1^, while BCF plants grew slower than WT plants under high-light (HL) conditions. Our results revealed that Chl *b* overproduced tomato plants could better adapt to LL conditions by absorbing more light for photosynthesis but adapt poorly to excess light conditions by accumulating more ROS and fewer anthocyanins. Enhanced production of Chl *b* is able to improve the growth rate of tomatoes that are grown under LL conditions, indicating the prospect of employing Chl *b* overproduced light-loving crops and ornamental plants for protected or indoor cultivation.

## 1. Introduction

Tomato (*Solanum lycopersicum* Mill.) is one of the widely cultured vegetables under protected cultivation, and it is a heliophile. Usually, the optimum irradiance of tomatoes is 500–800 µmol photons m^−2^ s^−1^, and the optimal light intensity requirement for growing tomatoes indoors is 400–500 µmol photons m^−2^ s^−1^ [1]. Because of covering frameworks under greenhouses and poor weather in spring and winter, the light intensity in facilities can be as weak as 10% of natural light [2]. Low-light (LL) stress becomes one of the primary factors limiting tomato growth and development under protected cultivation [3]. LL stress results in tomato plants with longer internodes, less vigorous growth, and low photosynthetic efficiency [3]. Their flower bud development and fruit set are also reduced in LL conditions. Additionally, earlier leaf senescence occurs in LL-grown tomatoes. Therefore, under LL conditions, the yield of tomatoes is significantly reduced. Creating new tomato germplasm that grows better under LL conditions is extremely urgent for improving the yield of tomatoes. It is also important to save the energy used to supplement light when the tomato is grown under protected cultivation.

Light intensity is a major environmental factor for photosynthesis and plant growth. Under LL conditions, large supercomplexes are formed by increasing the amounts of light harvest complex II (LHCII) to capture more energy for increased photosynthesis, resulting in better adaptation to the limited light conditions. Contrarily, under high-light (HL) conditions, a portion of the absorbed light energy is not used for photosynthesis. The over-excitation of the photosystem produces reactive oxygen species (ROS), which damages photosynthetic proteins and other functional proteins in chloroplasts. To prevent photodamage, a certain amount of LHCII will be degraded to reduce the light-harvesting capacity of photosystems [4]. The regulation of the construction and destruction of the LHC is one of the most important mechanisms for plants adapting to the light environment.

Chlorophyll (Chl) is the major pigment used by plants for capturing light energy. Higher plants possess two kinds of Chl: Chl *a* and Chl *b*. Chl *a* exists in both the core and LHC of the photosystem, while Chl *b* exclusively exists in LHC [5]. Chl *a* is essential for photochemistry, while Chl *b* provides plants an advantage in harvesting light around 450 nm, a wave region of light that is not efficiently absorbed by Chl *a*. Therefore, Chl *b* is highly important in increasing light harvesting in addition to being essential for stabilizing major light-harvesting Chl-binding proteins [6]. Thus, the synthesis and degradation of Chl *b* regulate the construction and destruction of the LHC [7].

Chlorophyllide *a* oxygenase (CAO) is the sole enzyme that converts Chl *a* to Chl *b* for Chl *b* biosynthesis. Chl *b* synthesis partly depends on the chlorophyllide *a* oxygenase (CAO) mRNA level. Increasing CAO mRNA synthesis by the cauliflower mosaic virus 35S promoter enhances Chl *b* accumulation [8]. The CAO mRNA level is immediately downregulated when plants are transferred from LL to HL conditions. In contrast, the CAO mRNA level gradually increases when HL-grown plants are transferred to LL conditions [9,10,11]. Chl *b* and LHC content are also regulated by the stability of the CAO protein level. CAO consists of three domains, designated as the A, B, and C domains. The C domain (C-terminal domain) catalyzes the conversion of Chl(ide) *a* to Chl(ide) *b.* The B domain links the A domain (N-terminal domain) and the C-domain. The A-domain destabilizes the CAO protein in the presence of sufficient Chl *b* [12,13]. The protease recognition sequence (degron) presents in this domain [14], and Clp protease involves in the degradation of CAO [15]. Transgenic plants expressing CAO B and C domains have shown a much higher Chl *b* level and lower Chl *a/b* ratio than WT plants [16]. Interestingly, no visible phenotypic change has been observed between the BC overexpressed plants and the WT plants when they were grown under LL conditions [16]. While transgenic plants expressing the full length of CAO do not accumulate CAO protein, and their Chl *b* level is similar to that of WT [17].

In this report, to examine the light adaptation of transgenic tomatoes with the enhanced production of Chl *b*, the B and C domains of Arabidopsis CAO were overexpressed in the tomato cultivar ‘Zhongshu-4’. The results showed that the overexpression of B and C domains of CAO led to increased Chl *b* synthesis and decreased the Chl *a/b* ratio. Compared with WT, Chl *b* enhanced, and the produced plants showed a faster growth rate under LL conditions, while they showed a slower growth rate under HL conditions. Our data support that the enhanced production of Chl *b* can be an effective strategy to improve the adaptation of tomatoes to LL conditions, one of the major environmental factors that limit tomato growth in protected cultivation. 

## 2. Results

### 2.1. BCF Was Overexpressed in Transgenic Tomato Plants 

**Figure 1:**
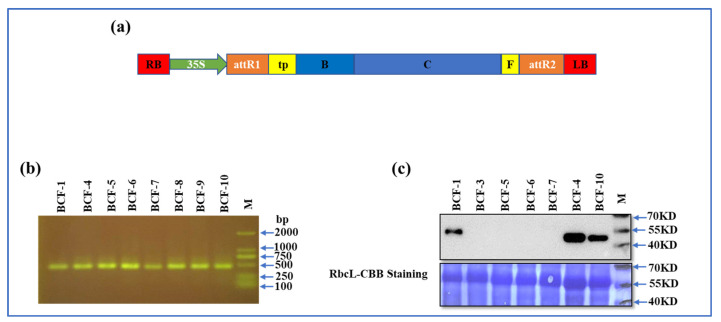
Vector construction and identification of transgenic plants expressing BCF. (**a**) Schematic presentation of the domain structures of the transgenic plants expressing *BCF*: The *B* and *C* domains of *CAO*, the predicted transit peptide (*TP*) sequence, and *FLAG* tag. (**b**) The 35S promoter and *BCF* inserted into the genome of the tomato plants were confirmed by PCR analysis, a target fragment of 477 bp. (**c**) The identification of the expression of BCF protein in positive transgenic plants by Western blotting. Anti-FLAG antibody was used as the primary antibody, and HRP goat anti-mouse IgG was used as the secondary antibody.

A truncated version of *CAO* (missing A domain) with a FLAG tag named *BCF* was overexpressed in the tomato cultivar ‘Zhongshu-4’ (Figure 1a). The transgenic plants that survived in kanamycin contained 1/2 MS medium and were identified by PCR first (Figure 1b). The results showed that all tested plants contained the 35S: *BCF* construct. The overexpressed BCF was further confirmed by immunoblotting. The results showed that transgenic lines BCF-1, BCF-4, and BCF-10 accumulated BCF protein (Figure 1c).

### 2.2. Physiological Characters of BCF Overexpressing Tomato Plants

**Figure 2:**
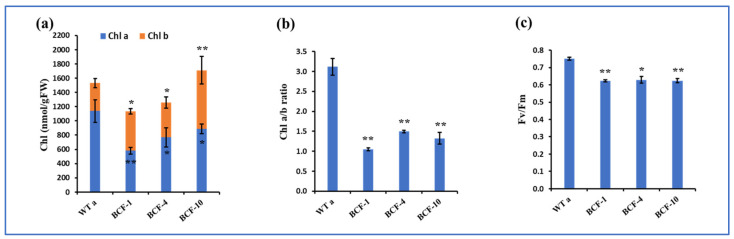
Physiological characteristics of WT and BCF-OE tomato plants. (**a**) Chlorophyll content (Chl *a* and Chl *b*). (**b**) Chl *a/b* ratio. (**c**) *Fv/Fm* of WT and BCF-OE lines. Each data point is the average of five biological replicates (five leaf tissues), and SE represents the standard error. Asterisks indicate a significant difference compared to WT tomato (Student’s *t*-test, * *p* < 0.05, ** *p* < 0.01).

Transgenic plants and nontransgenic segregant (WTa) grown under long-day conditions with normal light were analyzed. The results showed that BCF-1, BCF-4, and BCF-10 possessed a lower Chl *a* content than WTa, while the Chl *b* content of all the BCF-OE lines was significantly higher than that of the WTa plants. Chl *b* in BCF-OE lines was significantly higher than in WTa (Figure 2a). The Chl *a/b* ratio of the BCF-OE lines was almost decreased by 60% compared to WTa (Figure 2b). The maximum quantum efficiency of PSII photochemistry (*Fv/Fm*) was measured (Figure 2c). The results showed that the *F_V_/Fm* of WTa was higher than that in the BCF-OE lines, indicating the maximum photosynthetic potential of the BCF-OE plants was reduced.

### 2.3. BCF Overexpressing Plants Grow Better Than WT under LL Conditions

**Figure 3:**
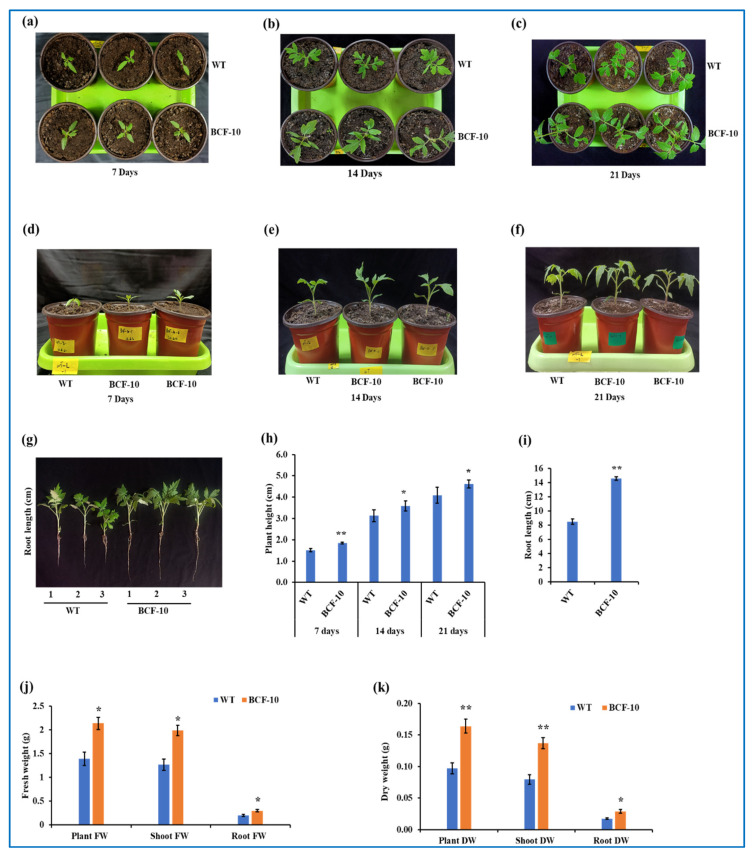
Morphological characterization of WT and BCF-OE plants under LL conditions. (**a**–**c**), Images showing the WT and BCF-OE plants that were grown under LL conditions for seven, fourteen, and twenty-one days. (**d**–**f**) Plant height of WT and BCF-OE plants at different LL treatment stages. (**g**) WT and BCF-OE plants’ root length after twenty-one days at LL treatment. (**h**) Plant height. (**i**) Root length. (**j**) Shoot, root, and total plants fresh weight of WT and BCF-OE plants. (**k**) Shoot, root, and total plants’ dry weight after being grown under LL conditions for three weeks. The data point averages six replicates, and SE represents standard error. Asterisks indicate a significant difference compared to WT (Student’s *t*-test, * *p* < 0.05, ** *p* < 0.01).

To examine whether BCF-OE plants can better adapt to LL conditions compared to WT plants, seven-day-old seedlings were transferred into LL conditions (50–70 µmol photons m^−2^ s^−1^). The data were recorded at seven, fourteen, and twenty-one days after the plants were transferred to LL growth conditions. The phenotypical results showed that BCF-OE plants grew better than WT under LL conditions (Figure 3a–c). The height of the BCF-OE plants was significantly increased compared with that of the WT plants (Figure 3d–h). Root length, plant fresh weight, shoot fresh weight, and root fresh weight were all significantly increased in the BCF-OE plants compared to the WT (Figure 3i,j). The plant DW, shoot DW, and root DW were increased by 40%, 42%, and 44% in BCF-OE plants compared to WT, respectively (Figure 3k).

**Figure 4:**
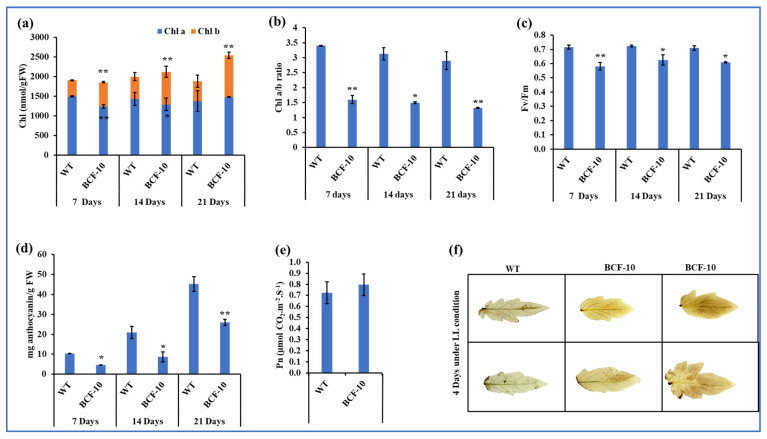
Pigments, photosynthetic rate, and H_2_O_2_ accumulation of WT and BCF-OE plants under LL conditions. (**a**) Chl *a* and Chl *b* content, (**b**) Chl *a/b* ratios, (**c**) Fv/Fm values, and (**d**) anthocyanin content of WT and BCF-OE plants in different stages of LL treatment. (**e**) Net photosynthetic rate (Pn) was measured after 14 days at LL growth conditions. (**f**) H_2_O_2_ accumulation, detached leaves of WT, and BCF-OE plants were exposed to LL for 2 and 4 days and stained with 3,3-diaminobenzidine (DAB). The data point is the average of six replicates, and SE represents the standard error. Asterisks indicate a significant difference compared to WT (Student’s *t*-test, * *p* < 0.05, ** *p* < 0.01).

The Chl *a* content of BCF-OE was significantly decreased compared to that of WT after seven days and fourteen days. More Chl *b* content was synthesized in the BCF-OE plants, which was 33.7%, 31%, and 52.2% higher than in the WT (Figure 4a). With the increased Chl *b*, the Chl *a/b* ratio of BCF-OE plants significantly decreased by more than 50% compared to that of the WT plants at all three stages (Figure 4b). The *Fv/Fm* value of BCF-OE plants was significantly lower than that of WT plants (Figure 4c and Figure S1), suggesting that the maximum photosynthetic potential of BCF-OE plants was also reduced under LL conditions. However, the net photosynthetic rate of the BCF-OE plants was slightly higher than that of the WT plants under LL conditions, although the statistical analysis results showed that the difference was not significant (Figure 4e). 

Additionally, we also found that BCF-OE plants accumulated less anthocyanin than WT plants under the same conditions (Figure 4d). Further, we investigated the production of H_2_O_2_ content by DAB staining. The results showed that BCF-OE plants stained slightly darker than WT leaves (Figure 4f). 

### 2.4. Phenotypic and Chl Metabolic Characterization of WT and BCF-OE Plants under HL Conditions

**Figure 5:**
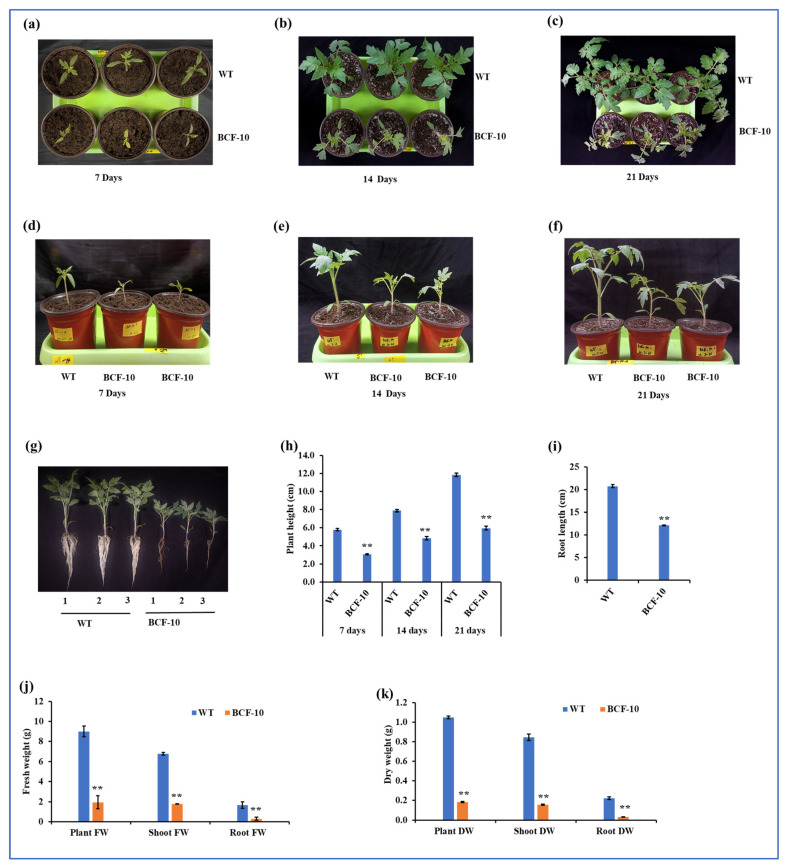
Phenotypical and morphological characterization of WT and BCF overexpression plants under HL growth conditions. (**a**–**c**) Images showing the growth of WT and BCF-OE tomato plants grown at HL growth conditions for 7, 14, and 21 days. (**d**–**f**) Plant height of WT and BCF-OE plants. (**g**) Root and shoot growth of 21-day-old WT and BCF-OE plants. (**h**) Plant height measurements. Plant height was measured from the base of the stem to the top of the plant. (**i**) Twenty-one-day-old plant root length, (**j**) plant fresh weight, shoot fresh weight, and root fresh weight, respectively. (**k**) Measurement of plant dry weight, shoot dry weight, and root dry weight. The data point is the average of six replicates, and SE represents the standard error. Asterisks indicate a significant difference compared to WT (Student’s *t*-test, ** *p* < 0.01).

We examined WT and BCF-OE plants grown under HL conditions (800–1000 µmol photons m^−2^ s^−1^). The phenotypes showed that HL strongly affected the growth of BCF-OE seedlings. BCF-OE seedlings showed a slower growth rate with shortened plant height compared to WT plants (Figure 5a–f). Seven-day-old seedlings were transferred to HL conditions for 7, 14, and 21 days’ of HL treatment. The average plant height of the BCF-OE plants was significantly reduced compared to the WT plants (Figure 5g,h). Additionally, root length, plant FW, shoot FW, and root FW were all significantly decreased in BCF-OE plants compared to WT plants (Figure 5i,j). The plant DW was 78.2%, shoot DW 81.5%, and root DW 85.44% reduced in BCF-OE plants relative to WT plants (Figure 5k). 

In order to investigate whether the different development stages of plants affect the growth rate of plants under HL treatment, BCF and WT plants were grown under normal light conditions for 20 days and were subsequently transferred into HL conditions. After HL treatment for 7, 14, and 21 days, we found that the size and height of the BCF and WT plants were similar (Figure S2). In addition, we found that the lateral branches of the BCF plants developed much faster than that of the WT during the same HL treatment (Figure S3).

**Figure 6:**
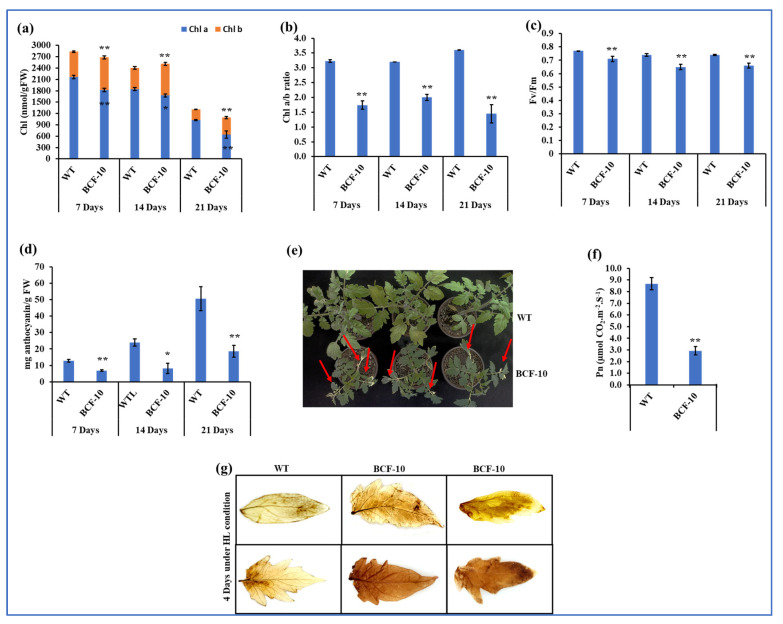
Chl metabolic characters, photosynthetic rate, and H_2_O_2_ accumulation of WT and BCF-OE plants under HL growth conditions. (**a**) Chl *a* and Chl *b* content of WT and BCF-OE plants. (**b**) Chl *a/b* ratio. (**c**) Fv/Fm values of 7, 14, and 21 days of HL. (**d**) Anthocyanin accumulation. (**e**) BCF-OE plants photodamaged under HL growth conditions. The red arrow shows the plant’s photodamaged. (**f**) Net photosynthetic rate (Pn) was measured after 14 days at HL growth conditions. (**g**) H_2_O_2_ accumulation of HL-grown WT and BCF-OE plants detached leaves were exposed to HL for 2 and 4 days and stained with 3,3-diaminobenzidine (DAB). The data point is the average of five replicates, and SE represents error bars. Asterisks indicate a significant difference compared to WT (Student’s *t*-test, * *p* < 0.05, ** *p* < 0.01).

Under HL conditions, the BCF-OE plants accumulated significantly less Chl *a* and more Chl *b* than the WT (Figure 6a and Figure S2). The Chl *a/b* ratio significantly decreased in BCF-OE plants during HL treatment compared to WT (Figure 6b). With HL treatment, both Chl *a* and Chl *b* content decreased. The Chl content significantly decreased under HL growth conditions, which raises the possibility of photodamage in BCF-OE plants (Figure 6e). The Fv/Fm value of BCF-OE plants was significantly lower than that of WT plants under HL growth conditions (Figure 6c). We also noted that BCF-OE plants produced significantly less anthocyanin than WT plants (Figure 6d). The net photosynthetic rate of BCF-OE plants was significantly decreased by 66.12% compared to WT under HL conditions (Figure 6f). Further, to investigate whether more ROS were produced due to the photodamage of BCF-OE plants, we examine H_2_O_2_ accumulation by DAB staining (Figure 6g). After two days and four days of HL treatment, BCF-OE leaves exhibited a strong brown color; at the same time, WT leaves were only slightly stained after four days of HL treatment. 

## 3. Discussion

Tomatoes are light-loving plants, and the optimal light intensity requirement for growing tomatoes indoors is 400–500 µmol photons m^−2^ s^−1^ [1]. In many cases, LL stress occurs in tomatoes under protected cultivation conditions. In this study, BCF overexpressed tomato plants showed significantly more Chl *b* content and lower Chl *a/b* ratios and Fv/Fm values compared to that of WT. These results were consistent with previous reports in which the BC domain of CAO was overexpressed in Arabidopsis [16]. It suggested that CAO protein levels should be well-regulated to avoid excess accumulation. The balance of Chl *a* and Chl *b* was stopped by the continuous expression of the catalyzation region of CAO protein, which could not be degraded in time. The BC domain of Arabidopsis CAO catalyzed the conversion of Chl *a* to Chl *b* in tomatoes as well. The low *Fv/Fm* value of the tomato BCF plants indicates that some overproduced Chl *b* molecules may be incorporated into Chl *a*-binding sites in the core complexes of photosystems, causing the energy transfer rates to decrease in BCF plants, similar to what happened to BC-overexpressed Arabidopsis [16,18]. 

Chl *b* overproduced plants were predicted to construct more LHC complexes in their photosystems, which can absorb more light [7]. Indeed, more LHC was formed in Arabidopsis expressing prokaryotic CAO (without A domain) or overexpressing BC of AtCAO [16,18]. Therefore, it was thought that Chl *b* overproduced plants would grow better than WT under light-limited conditions because more LHC can harvest more light. Surprisingly, Chl *b* overproduced Arabidopsis plants and WT plants shared a similar growth rate under LL conditions (70 µmol photons m^−2^ s^−1^) [18]. In this study, BCF plants accumulated more biomass than WT plants after they were transferred to LL conditions (Figure 3). Although the photosynthetic rate of BCF plants was slightly higher than that of WT (Figure 4), which could be the reason BCF plants grew faster than WT under LL conditions by the accumulation of biomass day by day. What caused the different growth performance between the Chl *b* overproduced Arabidopsis and the tomato? A light intensity of 130–150 µmol photons m^−2^ s^−1^ has been recommended for WT Arabidopsis growth [19], while 400–500 µmol photons m^−2^ s^−1^ is required for growing tomatoes indoors. Therefore, we suggested that the LL conditions for Arabidopsis were not low enough in the previous report. The light intensity of LL conditions was half of that for their normal growth, while for tomatoes in this study; the ratio was 1/10. Compared to WT plants, BCF plants could absorb more light for photosynthesis and growth; therefore, they can better adapt to extreme LL conditions. This study implied that Chl *b* overproduced light-loving plants could better adapt to extreme LL conditions by harvesting more light. 

Under HL conditions, the seven-day-old BCF seedlings grew slower and accumulated less biomass than the WT plants (Figure 5), while the size and height of BCF and WT plants were similar when twenty-day-old plants were treated with HL (Figure S2). Previous studies have shown that there were no significant differences in the fresh weights of rosette leaves between PhCAO (Chl *b* overproduced by overexpressing prokaryotic CAO) and WT Arabidopsis plants during HL acclimation [18]. In that study, twenty-five-day-old Arabidopsis plants were transferred to HL conditions for HL acclimation. Therefore, we infer that the development stage of plants is an important factor for investigating the growth rate of plants under HL conditions. The twenty-five-day-old Arabidopsis plants almost finished their vegetative growth phase; although HL conditions suppress the photosynthesis of Chl *b* overproduced plants, the fresh weight of mature rosette leaves was not much affected. Young seedlings require photosynthesis for rapid growth. The seven-day-old BCF seedlings could absorb more light energy than WT seedlings of the same age, but they could not use the energy for photosynthesis because they had low Fv/Fm values. On the contrary, excess energy is a large problem causing increasing ROS in the chloroplast, which can damage photosystems and affect chloroplast retrograde signaling [20,21]. Indeed, more H_2_O_2_ was detected in BCF plants than in WT plants after HL treatment. Therefore, BCF seedlings accumulated less biomass after HL treatment than WT plants (Figure 5). The results from the studies in BCF plants under HL conditions suggested that Chl *b* overproduced plants should avoid growth under HL conditions; otherwise, biomass or yield will be affected.

Under HL conditions, the H_2_O_2_ content in BCF plants was much higher than that in WT plants, indicating oxidative stress was heavier in the BCF plants than in the WT (Figure 6). The results were consistent with the Chl *b* overproduced Arabidopsis plants [16]. In addition, we found photodamage was happening to BCF plants but not to WT plants (Figure 6e), consistent with previous studies in Arabidopsis [16,18]. These results suggest the possibility of BCF plants possessing an insufficient thermal dissipation ability to cope with the excess energy. Another possible reason explaining the photodamage in BCF plants under HL conditions is the deficiency of anthocyanins in BCF plants (Figure 6d). Anthocyanins can scavenge ROS to maintain plant photosynthetic capacity [22]. However, anthocyanin synthesis genes are suppressed in Arabidopsis BC plants [16]. The relationship between Chl *b* and anthocyanin biosynthesis is still largely unknown. Chloroplast retrograde signals to the nucleus may regulate anthocyanin synthesis in BCF plants; indeed, it was reported that the genome’s uncoupled-dependent signaling pathway coordinates plastid biogenesis with the synthesis of anthocyanins [23]. Interestingly, DAB staining also implied that the oxidative stress of the BCF plants was slightly higher than that of the WT plants under LL conditions (Figure 4f), which may be related to the low level of anthocyanins in BCF compared with that of WT plants, and also the incorrect incorporation of Chl *b* into core complexes of photosystems. Therefore, even in LL conditions, some light energy could not be used for photosynthesis, and ROS were produced because photoinhibition occurred in BCF plants. Further investigation is required to address the reason in detail.

Taken together, we conclude that tomatoes with enhanced production of Chl *b* grew better under LL conditions and adapted to the HL conditions more poorly than WT plants. Therefore, it is possible to create new tomato germplasms by enhanced production of Chl *b* for protected cultivation with LL conditions. However, these germplasms have disadvantages when they face HL conditions.

## 4. Materials and Methods

### 4.1. Plant Materials and Growth Conditions

The WT tomato cultivar “Zhongshu-4”, which is a widely grown cultivar that has been bred by the China Academy of Agriculture Sciences, was used in this study. The seeds were kindly provided by Biorun Biosciences Company, Wuhan, China. Seeds were germinated on moist filter paper first. The germinated seeds were subsequently planted in soil (German K brand peat soil) under long-day conditions (16 h light/8 h dark) with an LED fluorescent light at 140–190 µmol photons m^−2^ s^−1^ (normal light) at a constant temperature of 23 °C. After seven or twenty days, one set, more than five uniform plants of WT and transgenic plants, was respectively transferred to LL growth conditions (50–70 µmol photons m^−2^ s^−1^), and another set was moved to the HL (800–1000 µmol photons m^−2^ s^−1^) growth conditions. Afterward, the phenotypic characters, plant growth, plant height, Chl contents, and *Fv/Fm* ratios were recorded every seven days.

### 4.2. Construction and Tomato Transformation

The transit peptide (*TP*) sequence *B* and *C* domains of *AtCAO* were cloned from the full-length complementary DNA (cDNA) of WT Arabidopsis (Col-0). The *FLAG* fragment was synthesized directly. These fragments were fused in order (named *BCF*) by overlapping PCR using super-fidelity DNA polymerase (Vazyme, Nanjing, China). The overlapping product was introduced into the Gateway entry vector pENTR223.1 and subsequently introduced into the Gateway compatible binary vector pK7WG2 by LR reaction [24]. The construct was transformed into WT with the help of the agrobacterium strain GV3101 [25]. The primers AtCAO-74-F: 5′TCTCCAGAAAGAAGGGCGT3′ and AtCAO-892-R: 5′GCATCTTCTTACATTCTCCATCG 3′ were used to identify transgene insertion by PCR.

### 4.3. Chl Analysis

Small pieces of the largest three leaves of each plant were first collected and weighed. Chl was extracted by homogenizing leaf tissue with pre-cooled acetone [26]. The extracts were centrifuged for 5 min at 15,000 ×rpm 4 °C. The supernatant was then transferred to new tubes. The supernatant was next diluted to 80% acetone, and finally, absorbance was measured at 646.6 nm and 663.6 nm with a spectrophotometer. Dilution was necessary to ensure the optimum ranges of absorbance of the spectrophotometer. The concentrations of Chl *a*, Chl *b,* and Chl *a* + *b* were calculated using the equations [27].

Chl *a* = 12.25·A^663.6^ − 2.55·A^646.6^ (1)

Chl *b* = 20.31·A^646.6^ − 4.91·A^663.6^ (2)

Chl *a* + *b* = 17.76·A^646.6^ + 7.34·A^663.6^ (3)

### 4.4. Chl Fluorescence Measurements

The method for measuring Chl fluorescence was similar to the previously described method [28]. After 15 min of darkness adaptation at room temperature, the maximum photochemical efficiency of photosystem II (Fv/Fm) was measured using a PHOTOSYNQ (MAC ID 52:00:06 ed. Designed and assembled in the USA) (www.photosynq.com/hello accessed on 5 May 2022).

### 4.5. Immunoblot Analysis

The total protein was isolated from 2–4 mg of fresh leaves from the tomato plant using 10 volumes (*v*/*w*) of a protein extraction buffer containing 50 mM Tris-HCl (pH 8.0), 1.5% (*w*/*v*) dithiothreitol, 12% (*w*/*v*) sucrose (Suc), and 2% (*w*/*v*) lithium lauryl sulfate. Before SDS-PAGE separation, all samples were mixed with an equal amount of 2× urea buffer containing 10 mM Tris-HCl (pH 8.0), 10% (*w*/*v*) Suc, 2% (*w*/*v*) SDS, 1 mM EDTA, 4 mM dithiothreitol, a small amount of bromophenol blue, and 10 M urea and were electrophoresed on 14% polyacrylamide gel and electro-blotted to PVDF membranes (Bio-Rad). Samples were loaded on the same weight of fresh leaves. The Flag-fused target protein was detected using anti-FLAG (ABclonal, Wuhan, China) as the primary antibody, and for the secondary antibody, HRP goat anti-mouse IgG (ABclonal, Wuhan, China) was used. 

### 4.6. Biomass Measurements

Plant height was measured from the base of the stem to the top of the plant. After 21 days of LL and HL treatments, root length and lateral branches length were measured with a ruler, and the whole plant’s fresh weight, shoot fresh weight, and root fresh weight were measured by the direct weighting method [29]. After weighing, plants were dried at 100 °C for 30 min and then at 55 °C until a constant weight was achieved, which was considered as the dry weight. 

### 4.7. Quantification of Anthocyanin

Anthocyanin was measured according to the previously published method [30]. Here, 1–2 mm leaf pieces from the largest three leaves were harvested from LL or HL treated plants and immediately weighed (5–6 mg). Then the leaves were placed in 1.5 mL microcentrifuge tubes containing 350 µL of extraction buffer (18% 1-propanol, 1% HCl, and 81% distilled water). The tubes were kept in boiling water for 3 min and then incubated in the dark for 6 h at room temperature. After incubation, the samples were centrifuged at 12,000 rpm for 5 min. Then 300 µL of the supernatant was transferred to new tubes, bringing the final volume up to 600 µL by adding extraction buffer. The total amount of anthocyanin was quantified by a spectrophotometer and expressed using the following equation [31].

(Abs _535_–Abs _650_) per gram of fresh weight (FW).

### 4.8. Determination of Net Photosynthesis Rate (Pn)

The Pn of the leaves of four plants for each treatment was measured using a photosynthesis instrument (LC Pro-SD, ADC Bio scientific, Hoddesdon, UK) as previously described [32]. Photosynthetic photon flux density (PPFD) was set to measure at 900 µmol photons m^−2^ s^−1^, and the experimental conditions were set at a relative humidity of 60–70 ± 10%, a temperature of 23 ± 0.5 °C, and with ambient CO_2_ levels of ca.400 PPM.

### 4.9. Detection of ROS

Staining of 3,3′-Diaminobenzidine (DAB) was employed to detect the hydrogen peroxide (H_2_O_2_) content. The staining method was described previously with slight modification [33]. Leaflets from three plants were detached after LL and HL treatment, placed into the DAB staining solution (5 mM DAB-HCl, pH = 3.8), and vacuum infiltrated for 3 min. The treated material was incubated for 10 h, then boiled for 5 min in an acetic acid: glycerol: ethanol (1:1:3 [*v*/*v*/*v*]) solution. DAB formed a deep brown polymerization product upon a reaction with H_2_O_2_ in the presence of peroxidase [34]. A digital camera was employed to take the image.

### 4.10. Data Analysis

A data set was collected and processed, the experimental diagrams were prepared with MS Excel 365, and figures were assembled with MS PowerPoint 365. The images of leaves were taken using a digital camera and processed with Adobe Photoshop CS3 (Adobe, San Jose, CA, USA). Data were analyzed using a Student’s *t*-test. Statistically significant differences were categorized into * *p* < 0.05 and ** *p* < 0.01.

## Data Availability

Data presented in this study are available upon request from the corresponding author.

## Supplementary Materials

The following supporting information can be downloaded at: https://www.mdpi.com/article/10.3390/ijms24043377/s1.

## Abbreviations

BCF	Transgenic Plants Expressing BC Domains of CAO Fused with FLAG Tag.
CAO	Chlorophyllide *a* Oxygenase
Chl *a*	Chlorophyll *a*
Chl *b*	Chlorophyll *b*
Chl	Chlorophyll
DAB	3,3-Diaminobenzidine
DW	Dry Weight
FW	Fresh Weight
H_2_O_2_	Hydrogen Peroxide
HL	High Light
LL	Low Light
PAP	Production of Anthocyanin Pigment
PPFD	Photosynthetic Photon Flux Density
ROS	Reactive Oxygen Species
WT	Wild-type
