# Supplementary material for: Effect of the Enhanced Production of Chlorophyll b on the Light Acclimation of Tomato
Source: Int J Mol Sci. 2023 Feb 8;24(4):3377. doi: 10.3390/ijms24043377 (PMC9961381; doi:10.3390/ijms24043377)
Supplement: Supplementary file 1 [file ijms-24-03377-s001.zip › ijms-2167546-supplementary.pdf]

Supplementary materials

Effect of the Enhanced Production of Chlorophyll *b* on the Light Acclimation of Tomato

Imran Khan [1,2,3], Ahmad Zada [1,2,3], Ting Jia [1,*] and Xueyun Hu [1,2,3,*]

1 International Research Laboratory of Agriculture and Agri-Product Safety of the Ministry of Education of China, Yangzhou University, Yangzhou 225009, China
2 Laboratory of Plant Functional Genomics of the Ministry of Education, Yangzhou University, Yangzhou 225009, China
3 College of Bioscience and Biotechnology, Yangzhou University, Yangzhou 225009, China
* Correspondence: tingj2012@yzu.edu.cn (T.J.); xyhulab@yzu.edu.cn (X.H.)

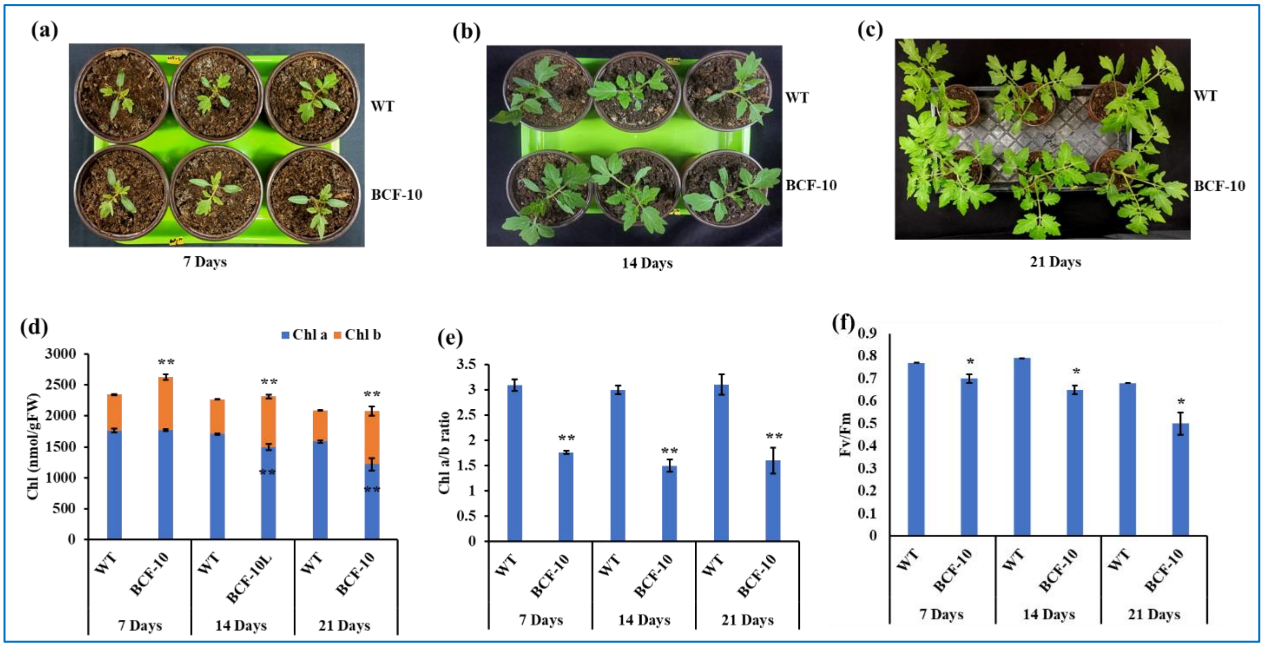

Figure S1. Morphological and Chl characterization of WT and BCF-OE transgenic tomato plants under LL conditions. The plants were grown in a growth chamber for 20 days. After 15 days, it was transferred to a LL growth condition. (**a-c**) plant phenotypes after 7, 14, and 21 days at LL condition. (**d**) Chl *a* and Chl *b* content of WT and transgenic plants (**e**) Chl *a*/*b* ratio (**f**) Fv/Fm values. The data point averages four replicates, and SE represents standard error. Asterisks indicate a significant difference compared to WT (Student's t-test, * $p < 0.05$, ** $p < 0.01$).

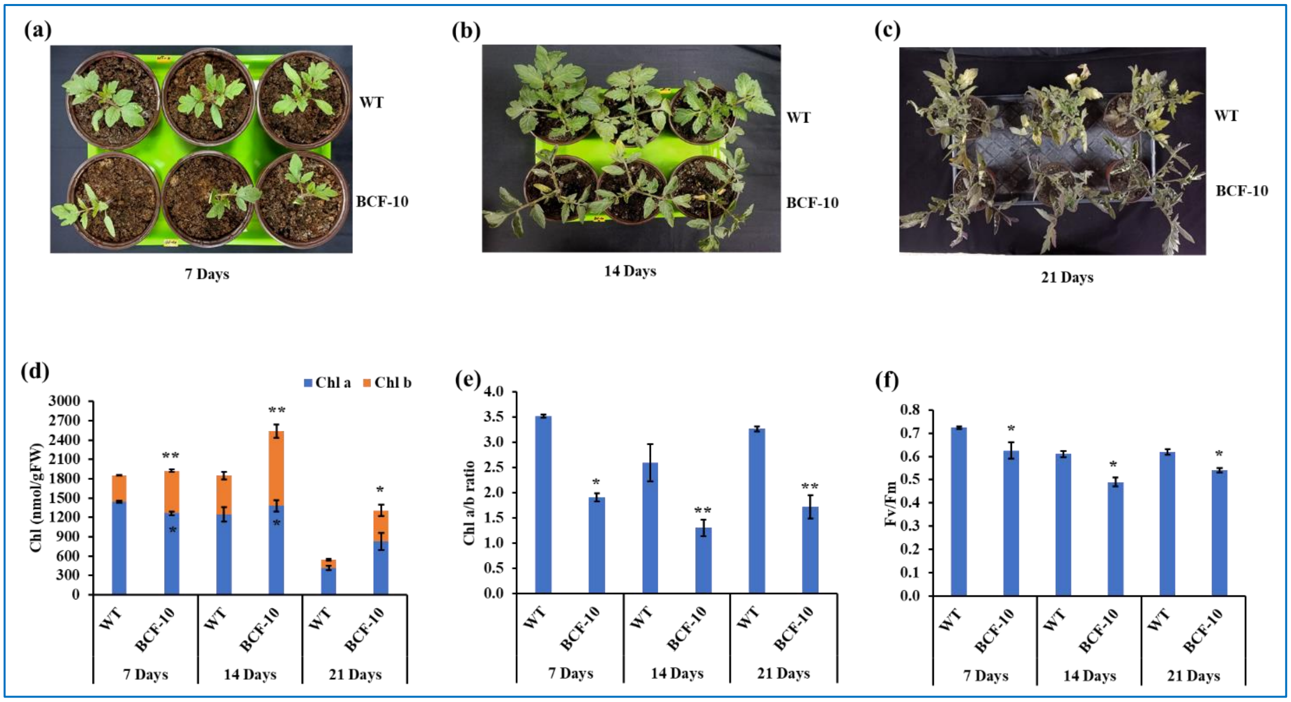

Figure S2. Phenotypes and physiological characterization of WT and BCF-OE transgenic tomato plants under HL-growth condition. The plants were grown in a growth chamber for 20 days. After 20 days, it was transferred to a HL growth condition. (**a-c**) phenotypes of WT and BCF-OE tomato plants. (**d**) Chl *a* and Chl *b* content, (**e**) Chl *a*/*b* ratio, (**f**) *Fv*/*Fm* values of WT and BCF-OE transgenic plants at HL condition. The data set averages four replicates, and SE represents the standard error. Asterisks indicate a significant difference compared to WT (Student's t-test, * $p < 0.05$, ** $p < 0.01$).

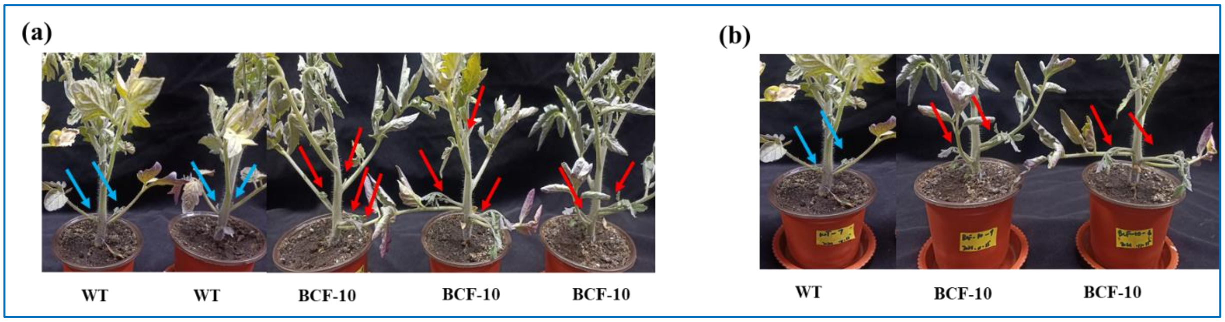

Figure S3. Lateral branches phenotypes of WT and BCF-OE tomato plants under HL condition. (**a,b**), Phenotypes of thirty-five days old tomato lateral branches. The blue arrow indicates the WT lateral branches, while the red arrows indicate BCF-OE lateral branches in the leaf axil.
